# Gigaxonin Potentiates Antiviral Innate Immune Responses by Targeting cGAS and TREX1

**DOI:** 10.1002/advs.202507146

**Published:** 2025-09-11

**Authors:** Ben‐Zhe Ji, Jie Wang, Yi Tu, Shan Zhang, Yong‐Sheng Wei, Yan He, Bin Zhang, Qing‐Qing Zhao, Hao‐Min Hu, Yu Liu

**Affiliations:** ^1^ College of Life Sciences State Key Laboratory of Virology and Biosafety Frontier Science Center for Immunology and Metabolism Hubei Key Laboratory of Cell Homeostasis Wuhan University Wuhan Hubei 430072 China; ^2^ School of Life Science and Technology Wuhan Polytechnic University Wuhan Hubei 430023 China

**Keywords:** cGAS, gigaxonin, innate immunity, interferon, TREX1

## Abstract

Innate immunity is the first line of defense against viral infections. Cyclic GMP‐AMP synthase (cGAS) senses abnormal cytosolic double‐stranded DNA (dsDNA) and triggers the production of interferon and proinflammatory cytokines to eliminate viruses, whereas three‐prime repair exonuclease 1 (TREX1) directly digests cytosolic dsDNA, thereby preventing aberrant activation of cGAS. The precise regulation of the antiviral response by cGAS and TREX1 remains incompletely understood. In this study, it is reported that gigaxonin potentiates antiviral innate immune responses by targeting both TREX1 and cGAS. Gigaxonin controls TREX1 turnover in the steady state by mediating its ubiquitination and proteasomal degradation. It also enhances the ubiquitination of cGAS and increases its enzymatic activity in response to infection with herpes simplex virus type 1 (HSV‐1). Furthermore, it is found that the binding of cGAS to gigaxonin is induced by HSV‐1 infection and that this interaction inhibits the TREX1‐gigaxonin interaction. The findings highlight the dynamic role of gigaxonin in enhancing antiviral innate immune responses by targeting both TREX1 and cGAS, suggesting that targeting gigaxonin may constitute a novel therapeutic approach for combating infectious diseases.

## Graphical abstract

TREX1 antagonizes DNA sensor cGAS by degrading cytosolic DNA. This work identifies gigaxonin as a molecular switch that coordinates DNA sensing and clearance pathways to amplify antiviral responses. Under the steady state, gigaxonin maintains cellular homeostasis by mediating TREX1 ubiquitination and proteasomal degradation. During DNA virus infection, gigaxonin binds with cGAS, enhances cGAS ubiquitination and enzymatic activation.

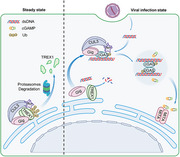

## 1 Introduction

The innate immune response plays a crucial role in defending against viral infection.Cytosolic cGAS is the primary DNA sensor that detects pathogenic dsDNA and leaked mitochondrial DNA upon viral infection.^[^ ^1^ ^]^ Activated cGAS catalyzes the synthesis of the second messenger, cyclic GMP‐AMP (cGAMP), which then binds to the ER‐localized mediator of interferon regulatory factor activation (MITA, also referred to as stimulator of interferon genes (STING)).^[^ ^2^, ^3^ ^]^ MITA recruits and activates the kinases TANK‐binding kinase 1 (TBK1) and IκB kinase, resulting in the phosphorylation and nuclear translocation of the transcription factors interferon regulatory factor 3 (IRF3) and NF‐κB.^[^ ^4^, ^5^, ^6^ ^]^ These transcription factors initiate the expression of type I interferons (IFNs) and other immunomodulatory proteins, which contribute to antiviral immune responses.^[^ ^7^, ^8^ ^]^ To date, cGAS‐mediated signaling has been implicated in various diseases, not only infectious and autoimmune diseases but also neurological diseases and cancers.^[^ ^9^, ^10^, ^11^, ^12^, ^13^, ^14^, ^15^ ^]^

In addition to the cGAS‐mediated innate immune response, cells possess a rapid mechanism for dealing with abnormal cytosolic DNA. TREX1 is an ER‐located exonuclease that digests both dsDNA and single‐stranded DNA, thereby preventing cGAS activation by degrading cytosolic DNA.^[^ ^16^, ^17^, ^18^ ^]^ *Trex1^−/−^* fibroblasts and macrophages express elevated interferon‐stimulated genes, whereas *Trex1*‐deficient or *Trex1*‐nuclease‐deficient mice display Aicardi‐Goutières syndrome (AGS)‐like phenotypes, which can be reversed by the deletion of *cGas*, *Mita* or *Ifnb1*.^[^ ^19^, ^20^ ^]^ Furthermore, multiple mutations within the *TREX1* gene result in cGAS‐driven immune activation and cause multiple type I IFN‐associated autoimmune diseases, such as AGS), familial chilblain lupus, and retinal vasculopathy with cerebral leukodystrophy.^[^ ^21^, ^22^, ^23^ ^]^ Therapeutic modalities that target TREX1, such as small‐molecule inhibitors, have been reported to be efficient for the treatment of tumors associated with cGAS.^[^ ^24^, ^25^ ^]^ Although the importance of TREX1 for health has been reported, how TREX1 is precisely regulated to maintain immune homeostasis is still largely unknown.

In this study, we identifies gigaxonin as a molecular switch that coordinates DNA sensing and clearance pathways to potentiate antiviral responses. Gigaxonin has been previously reported to be crucial for the turnover of neural intermediate filaments and the cytoskeleton.^[^ ^26^, ^27^, ^28^ ^]^ Mutations within the *gigaxonin* gene cause a severe inherited neurodegenerative disorder, giant axonal neuropathy.^[^ ^29^, ^30^, ^31^ ^]^ Although gigaxonin is widely distributed, its function beyond the nervous system remains unclear. By mass spectrum identification, we revealed the association of gigaxonin with cGAS upon HSV‐1 infection. We found that gigaxonin potentiates innate immune cGAS signaling by increasing the enzymatic activity of cGAS. Furthermore, gigaxonin mediates the ubiquitination of both cGAS and TREX1 by the cullin‐RING E3 ubiquitin ligase 3 (CRL3) complex, triggering cGAS activation upon virus infection and TREX1 degradation in the steady state. Our data reveal a novel mechanism by which gigaxonin affects both DNA sensor cGAS and DNA exonuclease TREX1, highlighting its critical role in modulating antiviral innate immune responses.

## 2 Results

### 2.1 Gigaxonin Potentiates Cellular Immune Responses to DNA Viruses

**Figure 1:**
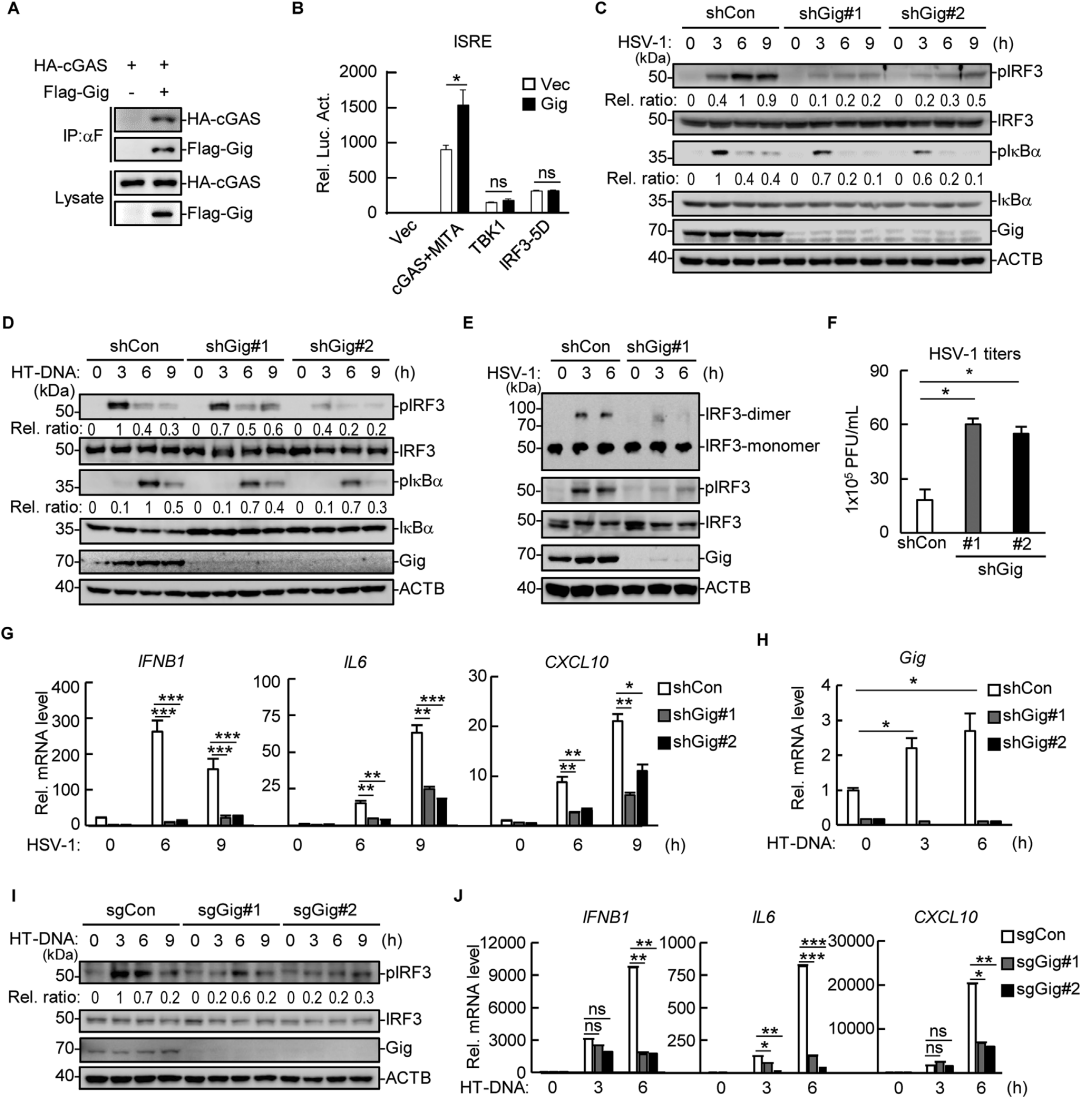
Gigaxonin potentiates cellular immune responses to DNA viruses. A) HEK293T cells were transfected with HA‐cGAS and Flag‐gigaxonin (Gig) before co‐IP and immunoblotting analysis with the indicated antibodies. B) HEK293T cells were transfected with the ISRE luciferase reporter (0.1 µg mL^−1^), TK‐*Renilla* luciferase reporter (0.1 µg mL^−1^), or the indicated plasmids (0.1 µg mL^−1^ each). Luciferase assays were performed 20 h after transfection. C,D) Specific shRNA plasmids #1 and #2 targeting *gigaxonin* and shRNA plasmid targeting the scramble sequence were transduced into THP‐1 cells to establish stable *gigaxonin*‐knockdown (shGig #1 and shGig #2) and control (shCon) THP‐1 cell lines. The cells were infected with HSV‐1 (MOI = 1) (C) or transfected with HT‐DNA (1 µg mL^−1^) (D) for the indicated times, followed by immunoblotting analysis. The phosphorylated bands were quantified using ImageJ software and normalized to the levels of the control protein β‐actin (ACTB). E) Stable *Gigaxonin*‐knockdown (shGig#1) and control THP‐1 cells were infected with HSV‐1 (MOI = 1) for the indicated times before Native‒PAGE (top panel) or SDS‒PAGE (bottom panel) was performed. F) Stable *Gigaxonin*‐knockdown and control THP‐1 cells were infected with HSV‐1 (MOI = 1) for 24 h before plaque assays were performed. G,H) Stable *Gigaxonin*‐knockdown and control THP‐1 cells were infected with HSV‐1 (MOI = 1) (G) or transfected with HT‐DNA (1 µg mL^−1^) (H) for the indicated times before qPCR analysis. I,J) *Gigaxonin*‐deficient (transfected with sgRNA targeting gigaxonin, marked as sgGig#1 and sgGig#2) and control (sgCon) THP‐1 cells were transfected with HT‐DNA (1 µg mL^−1^) for the indicated time, followed by immunoblotting (I) or qPCR (J). The phosphorylated bands were quantified and normalized to the levels of the control ACTB. The bar graphs show the mean ± SD of a representative experiment performed in triplicate. ^∗^ *p* < 0.05; ^∗∗^ *p* < 0.01; ^∗∗∗^ *p* < 0.001; ns, not significant.

To identify novel regulators involved in cGAS‐mediated innate immune responses, we performed co‐immunoprecipitation (co‐IP) in the murine macrophage line RAW264.7 using an anti‐mouse cGAS antibody in combination with mass spectrometry. Murine gigaxonin, characterized by 13 unique peptides, was identified in the HSV‐1‐infected group, with exceptional confidence among the candidates (Figure S1A, Supporting Information). We then confirmed that the overexpressed human homolog gigaxonin interacted with cGAS via co‐IP (**Figure** **1**A). Dual‐luciferase reporter assays revealed that gigaxonin promoted the activation of ISRE and IFNB induced by overexpressed cGAS and MITA but had little effect on downstream TBK1‐ or IRF3‐5D‐mediated ISRE and IFNB activation (Figure 1B; S1B, Supporting Information).

To determine the role of endogenous gigaxonin in cGAS‐mediated antiviral immune responses, we established a stable gigaxonin‐knockdown human monocytic leukemia cell line, THP‐1, via shRNA transduction and treated the cells with HSV‐1 infection or herring testis‐DNA (HT‐DNA) transfection. The results revealed that gigaxonin knockdown significantly attenuated the phosphorylation of IRF3 induced by HSV‐1 infection or HT‐DNA transfection and mildly attenuated the phosphorylation of IκBα (Figure 1C,D). Consistently, gigaxonin knockdown impaired HSV‐1‐induced dimerization of IRF3 (Figure 1E). HSV‐1 titers were also increased by gigaxonin knockdown (Figure 1F). HSV‐1‐ or HT‐DNA‐induced transcription of the downstream molecules *IFNB1*, *IL6*, and *CXCL10* was also dampened in gigaxonin‐knockdown cells (Figure 1G; Figure S1C; Supporting Information). Consistently, the transcription of *gigaxonin* markedly increased upon HT‐DNA transfection (Figure 1H). Similar results were obtained using gigaxonin‐deficient THP‐1 cells, which were generated via the CRISPR‐Cas9 technique (Figure 1I,J). These results suggest that gigaxonin potentiates cGAS‐mediated immune responses to abnormal cytosolic DNA. Because the effect of shRNA construct #1 was greater than that of shRNA construct #2, we selected the shGig#1 construct for the experiments described below unless otherwise noted.

### 2.2 The Mouse Homolog Gigaxonin Participates in dsDNA‐Induced Innate Immune Responses

**Figure 2:**
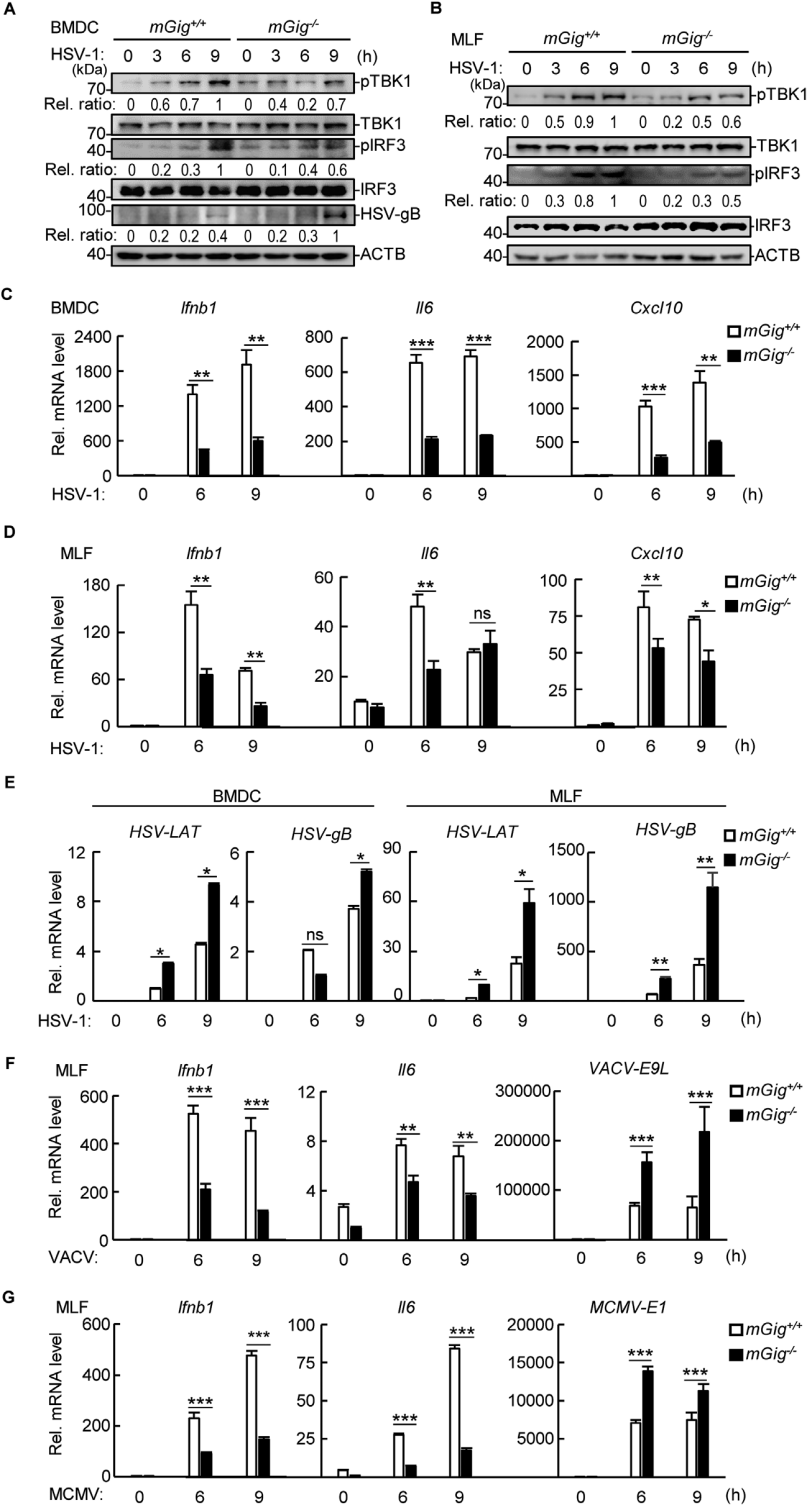
The mouse homolog gigaxonin participates in dsDNA‐induced innate immune responses. A,B) *mGig^+/+^* and *mGig^−/−^* BMDCs (A) or MLFs (B) were infected with HSV‐1 (MOI = 1) for the indicated times, followed by immunoblotting analysis. The phosphorylated bands were quantified and normalized to the levels of the control ACTB. C–E) *mGig^+/+^* and *mGig^−/−^* BMDCs (C, E) or MLFs (D, E) were infected with HSV‐1 (MOI = 1) for the indicated times, followed by qPCR analysis. F,G) *mGig* ^+/+^ and *mGig* ^−/−^ MLFs were infected with VACV (F, MOI = 10) or MCMV (G, MOI = 5) for the indicated times, followed by qPCR analysis. The bar graphs show the mean ± SD of a representative experiment performed in triplicate. ^∗^ *p* < 0.05; ^∗∗^ *p* < 0.01; ^∗∗∗^ *p* < 0.001; ns, not significant.

To explore whether mouse gigaxonin functions similarly in innate immunity, we isolated and cultured mouse bone marrow‐derived dendritic cells (BMDCs) and lung fibroblasts (MLFs) from *Gig* ^−/−^ mice (Figure S2A, Supporting Information). HSV‐1 infection induced robust phosphorylation of TBK1 and IRF3 in *Gig^+/+^* BMDCs and MLFs but much weaker phosphorylation in *Gig^−/−^* cells (**Figure** **2**A,B). In contrast, the level of the viral fusion protein glycoprotein B (gB) was much greater in *Gig^−/−^* BMDCs than in control BMDCs (Figure 2A). Consistently, the transcription of downstream *Ifnb1*, *Cxcl10*, and *Il6* was significantly suppressed in *Gig^−/−^* BMDCs and MLFs (Figure 2C,D). The transcription of HSV‐1 latency‐associated transcript (*LAT*) and *gB* was significantly increased in *Gig^−/−^* BMDCs and MLFs (Figure 2E). Similar results were obtained in MLFs infected with vaccinia virus (VACV) or murine cytomegalovirus (MCMV) (Figure 2F,G). These data suggested that mouse gigaxonin potentiated antiviral immune responses to DNA viruses.

It has been reported that HSV‐1 infection causes mitochondrial stress, which leads to the leakage of mitochondrial DNA (mtDNA) into the cytoplasm and subsequent activation of the cGAS pathway.^[^ ^32^ ^]^ We then treated MLFs with HT‐DNA or a combination of the BCL‐2 inhibitor ABT737 (5 µm), the MCL‐1 inhibitor S63845 (5 µm), and the pancaspase inhibitor z‐VAD (5 µm) to induce mtDNA leakage.^[^ ^33^, ^34^, ^35^ ^]^ The results from the qPCR analysis revealed that the HT‐DNA‐ or mtDNA‐induced transcription of *Ifnb1*, *Cxcl10*, and *Il6* was significantly suppressed by gigaxonin knockout (Figure S2B,C; Supporting Information). These data suggest that the immune function of mouse gigaxonin is dependent on the cGAS‐mediated signaling pathway.

### 2.3 Gigaxonin Potentiates cGAS Activity through the CRL3 Complex

**Figure 3:**
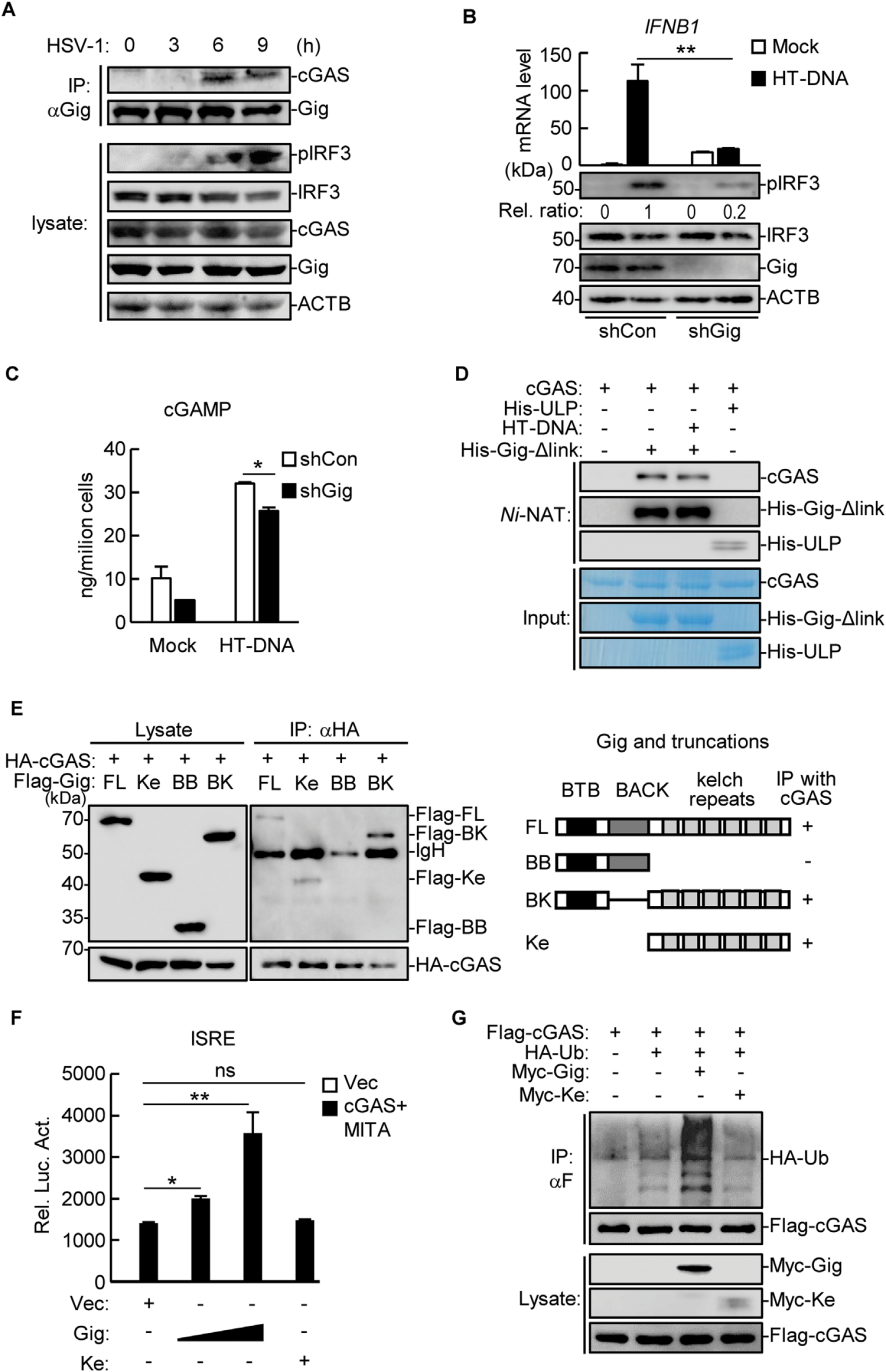
Gigaxonin potentiates cGAS activity through the CRL3 complex. A) THP‐1 cells were infected with HSV‐1 (MOI = 1) for the indicated times before co‐IP and immunoblotting analysis with the indicated antibodies. B) Stable *Gig*‐knockdown and control THP‐1 cells were transfected with HT‐DNA (1 µg mL^−1^) or left untreated (Mock) for 4 h before immunoblotting (bottom panel). The phosphorylated bands were quantified and normalized to the levels of the control ACTB. Cytosolic extracts were collected and incubated with digitonin‐pretreated HaCaT cells for 4 h, followed by qPCR analysis (top panel). The data represent the mean ± SD;^∗∗^ *p* < 0.01. C) Stable *Gig*‐knockdown and control THP‐1 cells were transfected with HT‐DNA (1 µg mL^−1^) or left untreated (Mock) for 4 h, followed by ELISA. The data represent the mean ± SD; ^∗^ *p* < 0.05. D) Recombinant cGAS and His‐Gig‐Δlink protein, or His‐ULP as a negative control, were mixed with or without HT‐DNA (10 µg mL^−1^) and incubated with *Ni‐NTA*‐Sepharose beads, followed by immunoblotting (top panel) or Coomassie blue staining (bottom panel). E) HEK293T cells were transfected with the indicated plasmids, followed by co‐IP and immunoblotting analysis (left panel). The results are also shown in the schematic diagram (right panel). FL, full length; BB, BTB and BACK domains; BK, BTB and Kelch repeats; Ke, kelch repeats. F) HEK293T cells were transfected with the ISRE luciferase reporter (0.1 µg mL^−1^), TK‐*Renilla* luciferase reporter (0.01 µg mL^−1^), cGAS (0.1 µg mL^−1^), MITA (0.1 µg mL^−1^), Gig (0.1 and 0.2 µg mL^−1^) and Kelch (0.1 µg mL^−1^). Luciferase assays were performed 20 h after transfection. The data represent the mean ± SD of a representative experiment performed in triplicate; ^∗^ *p* < 0.05; ^∗∗^ *p* < 0.01; ns, not significant. G) HEK293T cells were transfected with the indicated plasmids, followed by co‐IP and immunoblotting analysis.

To investigate how gigaxonin is involved in cytosolic dsDNA‐triggered signaling, we performed endogenous co‐IP and found that endogenous gigaxonin interacted with cGAS upon HSV‐1 infection (**Figure** **3**A). A cGAMP activity assay was subsequently performed to examine whether gigaxonin affects the activity of cGAS. Cell extracts from HT‐DNA‐treated gigaxonin‐knockdown or control THP‐1 cells were delivered to digitonin‐permeabilized HaCaT cells. The transcription of *IFNB1* in HaCaT cells was then tested to determine the presence of cGAMP in cell extracts from THP‐1 cells.^[^ ^3^, ^36^ ^]^ HT‐DNA transfection led to intensive phosphorylation of IRF3 in control cells but much weaker phosphorylation in gigaxonin‐knockdown cells (Figure 3B, bottom panel). As expected, cell extracts from stimulated control cells, but not stimulated gigaxonin‐knockdown cells, induced the transcription of *IFNB1* in permeabilized HaCaT cells (Figure 3B, top panel), suggesting that gigaxonin knockdown impairs the cGAS‐catalyzed production of cGAMP. Similar results were obtained via ELISA (Figure 3C).

To examine whether gigaxonin directly interacts with cGAS and regulates cGAS activity, we prepared recombinant proteins via a prokaryotic expression system. During the protein purification process, the recombinant full‐length gigaxonin was cleaved between the BACK domain and the kelch repeats (Figure S3A, Supporting Information). Therefore, His‐tagged gigaxonin truncation lacking those residues between the BACK domain and the kelch repeats (His‐Gig‐Δlink) was used instead. Recombinant cGAS was incubated with His‐Gig‐Δlink or control His‐tagged ubiquitin‐like protein‐specific protease (His‐ULP) and subjected to *Ni‐NTA* pull‐down assays. The results revealed that His‐Gig‐Δlink, but not His‐ULP, pulled down cGAS, suggesting that gigaxonin directly interacts with cGAS (Figure 3D). Additionally, supplementation with HT‐DNA had little effect on the interaction between cGAS and His‐Gig‐Δlink. Furthermore, in vitro cGAS activity assays revealed that supplementation with gradient amounts of His‐Gig‐Δlink did not markedly affect the cGAS‐catalyzed production of Pi triggered by HT‐DNA (Figure S3B, Supporting Information).^[^ ^36^ ^]^ These results from in vitro assays suggest that gigaxonin directly binds to cGAS but cannot regulate cGAS activity alone.

Because gigaxonin is reported to function as a substrate‐recognizing module of the CRL3 complex, we then performed co‐IP for domain mapping of the interaction between gigaxonin and cGAS. The results revealed that cGAS interacted with the kelch repeats, but not the BTB or BACK domains, of gigaxonin (Figure 3E). Dual luciferase reporter assays revealed that the kelch repeats of gigaxonin had little effect on the cGAS signaling‐induced activation of ISRE (Figure 3F), suggesting that the ability of gigaxonin to potentiate cGAS activation is dependent on the gigaxonin‐CRL3 E3 ligase complex. Consistently, full‐length gigaxonin, but not kelch repeats, triggered the ubiquitination of cGAS without affecting cGAS expression (Figure 3G). These results indicated that gigaxonin potentiates cGAS activation by mediating CRL3‐catalyzed cGAS ubiquitination.

### 2.4 Gigaxonin Increases Abnormal Cytosolic DNA Levels by Mediating TREX1 Degradation

**Figure 4:**
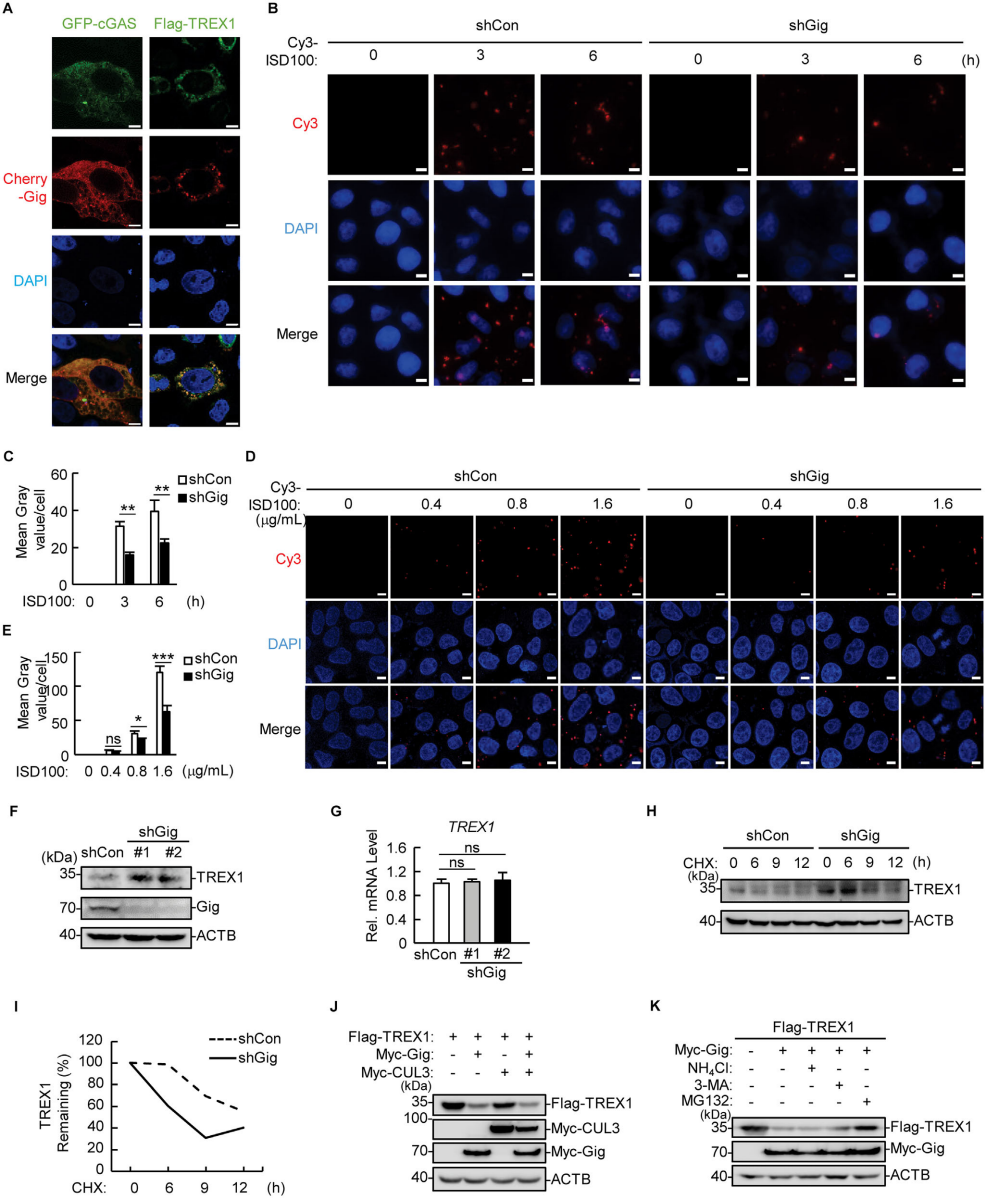
Gigaxonin increases abnormal cytosolic DNA levels by mediating the degradation of TREX1. A) HeLa cells were transfected with Cherry‐Gig, Flag‐TREX1 or GFP‐cGAS before confocal microscopy observation. Scale bars, 10 µm. B,C) Stable *Gig*‐knockdown and control HeLa cells were transfected with Cy3‐tagged ISD100 (0.7 µg mL^−1^) for the indicated times and visualized via confocal microscopy (B). Scale bars, 10 µm. The fluorescence intensity of Cy3 from six visual fields of each group was measured, and the average Cy3 intensity per cell was analyzed via ImageJ (C). The data represent the mean ± SD derived from six visual fields of a representative experiment; ^∗∗^ *p* < 0.01. D,E) Stable *Gig*‐knockdown (shGig) and control HeLa cells were transfected with the indicated amount of Cy3‐tagged ISD100 for 6 h and visualized via confocal microscopy (D). Scale bars, 10 µm. The average Cy3 intensity per cell was analyzed via ImageJ (E). The data represent the mean ± SD derived from six visual fields of a representative experiment; ^∗^ *p* < 0.05; ^∗∗∗^ *p* < 0.001; ns, not significant. F,G) Stable *Gig*‐knockdown and control HeLa cells were analyzed by immunoblotting (F) or qPCR (G). H,I) Stable *Gig*‐knockdown and control HeLa cells were treated with cycloheximide (CHX, 50 µg mL^−1^) for the indicated times, followed by immunoblotting analysis (H). The relative amount of TREX1 remaining is shown in Figure I.J) HEK293T cells were transfected with Myc‐Gig, Myc‐CUL3, or Flag‐TREX1, followed by immunoblotting analysis. K) HEK293T cells were transfected with Myc‐Gig or Flag‐TREX1 for 18 h and then treated with MG132, NH_4_Cl, or 3‐MA for 6 h before immunoblotting.

Colocalization studies were then performed via confocal microscopy. Surprisingly, gigaxonin colocalized with not only cGAS but also TREX1 (**Figure** **4**A). As the major cytosolic DNA exonuclease, TREX1 accounts for 60% to 70% of the total cellular exonuclease activity.^[^ ^18^ ^]^ Therefore, we transfected Cy3‐tagged 100‐bp immune‐stimulatory DNA (ISD100) into stable gigaxonin‐knockdown or control cells. By monitoring the intensity of Cy3 fluorescence at 3 and 6 h after transfection, the Cy3 fluorescence in the gigaxonin‐knockdown cells was significantly weaker than that in the control cells at different time points after stimulation (Figure 4B,C). When we transfected different amounts of Cy3‐ISD100 into cells, differences in Cy3 fluorescence were observed between the gigaxonin‐knockdown and control cells in a dose‐dependent manner, suggesting that gigaxonin increases the amount of cytosolic dsDNA (Figure 4D,E).

We then sought to explore whether gigaxonin functions through interference with TREX1. Gigaxonin knockdown resulted in a marked increase in the amount of endogenous TREX1 in HeLa cells (Figure 4F), whereas the TREX1 mRNA level was not significantly changed by gigaxonin knockdown (Figure 4G). Cycloheximide chase assays revealed that the degradation of endogenous TREX1 was markedly delayed by gigaxonin knockdown, suggesting that gigaxonin is highly relevant to the stability of TREX1 (Figure 4H,I). Moreover, overexpression of gigaxonin, but not CUL3, significantly reduced the expression of Flag‐TREX1 (Figure 4J). Compared with transfection with gigaxonin alone, cotransfection of gigaxonin with CUL3 did not increase the reduction in TREX1, suggesting that endogenous CUL3 is sufficient to support gigaxonin‐mediated TREX1 degradation (Figure 4J).

To explore which pathway is responsible for gigaxonin‐induced degradation of TREX1, we treated cells with the proteasome inhibitor MG132, the autophagy inhibitor 3‐methyladenine (3‐MA), and the lysosome inhibitor NH_4_Cl. MG132 treatment, but not 3‐MA or NH_4_Cl treatment, rescued gigaxonin‐mediated TREX1 degradation (Figure 4K), suggesting that gigaxonin triggers the proteasomal degradation of TREX1.

### 2.5 Gigaxonin Promotes the Ubiquitination of TREX1

**Figure 5:**
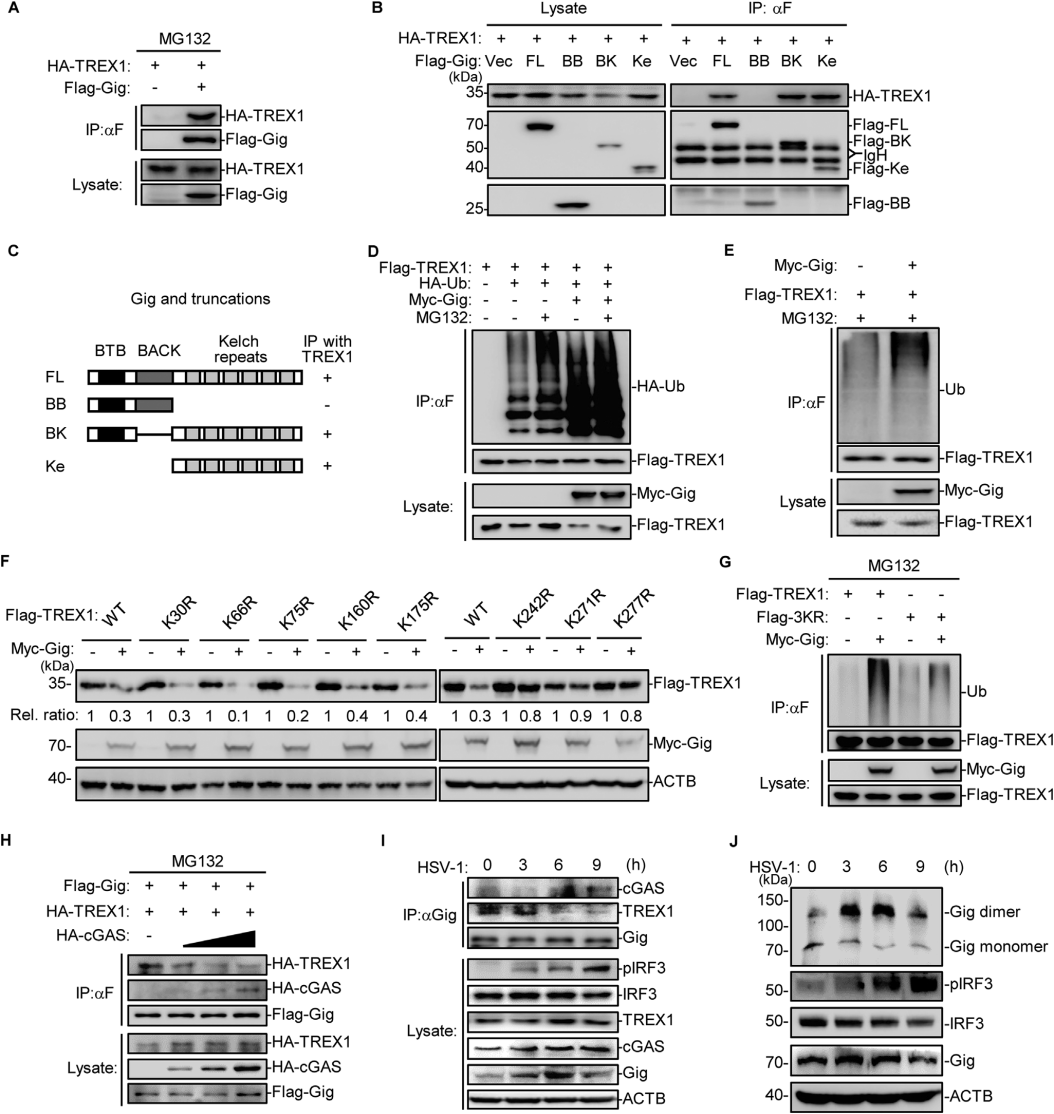
Gigaxonin promotes the ubiquitination of TREX1. A–C) HEK293T cells were transfected with the indicated plasmids and treated with MG132 for 6 h, followed by co‐IP and immunoblotting analysis (A, B). The domain mapping results are shown in the schematic diagram (C). D,E) HEK293T cells were transfected with the indicated plasmids and treated with or without MG‐132 for 6 h, followed by co‐IP and immunoblotting analysis. F) HEK293T cells were transfected with the indicated plasmids, followed by immunoblotting analysis. The Flag‐TREX1 bands were quantified and normalized to the levels of the control ACTB. G,H) HEK293T cells were transfected with the indicated plasmids and treated with MG‐132 for 6 h, followed by co‐IP and immunoblotting analysis. I) THP‐1 cells were infected with HSV‐1 (MOI = 1) for the indicated times, followed by co‐IP and immunoblotting analysis. J) THP‐1 cells were infected with HSV‐1 (MOI = 1) for the indicated times before native‒PAGE (top panel) or SDS‒PAGE (bottom panel) analysis.

To determine how gigaxonin triggers the degradation of TREX1, we performed co‐IP and confirmed the interaction between TREX1 and MG132 (**Figure** **5**A). Domain mapping revealed that gigaxonin interacted with TREX1 through its kelch repeats (Figure 5B,C).

We next examined whether gigaxonin mediates the ubiquitination of TREX1. As shown in Figure 5D, cotransfection of HA‐ubiquitin with TREX1 led to a decrease in TREX1 in cell lysates and detectable ubiquitination of TREX1. Adding MG132 rescued the expression of TREX1 and increased TREX1 ubiquitination. Cotransfection of Myc‐gigaxonin with or without HA‐ubiquitin markedly increased TREX1 ubiquitination, which was increased by MG132 (Figure 5D,E). To identify which lysine residue of TREX1 is required for gigaxonin‐mediated degradation, we generated TREX1 point mutants by replacing each lysine residue with an arginine residue. The TREX1 mutants K242R, K271R, and K277R partially resisted gigaxonin‐mediated degradation (Figure 5F). We then mutated these three lysines to arginines (TREX1–3KR) and found that the gigaxonin‐mediated ubiquitination of TREX1–3KR was much weaker than that of wild‐type TREX1 (Figure 5G). These results demonstrated that gigaxonin promotes the degradation of TREX1 through mediating the ubiquitination of TREX1 at three specific lysines via the CRL3 complex.

Because gigaxonin interacts with both cGAS and TREX1 through its kelch repeats, we further investigated how gigaxonin interacts with both cGAS and TREX1 in cells. Transfection with HA‐cGAS attenuated the interaction between gigaxonin and TREX1 in a dose‐dependent manner (Figure 5H). Endogenous co‐IP analysis revealed that gigaxonin interacted with TREX1 in the steady state, whereas the endogenous interaction between gigaxonin and cGAS upon stimulation attenuated the interaction between gigaxonin and TREX1 (Figure 5I). Because both TREX1 and cGAS function as obligate homodimers, we then performed native PAGE to test whether gigaxonin forms dimers as well. As shown in Figure 5J, gigaxonin existed as both dimer and monomer in the steady state, and HSV‐1 infection promoted the dimerization of gigaxonin.

These results suggest that gigaxonin maintains the basal level of TREX1 in the steady state by mediating the turnover of TREX1 through the CRL3 complex. Moreover, cGAS interacts with gigaxonin upon stimulation, which prevents TREX1 from approaching gigaxonin through competitive interactions with the same conserved domain.

### 2.6 Gigaxonin is Required for Host Defense Against DNA Viruses in Mice

**Figure 6:**
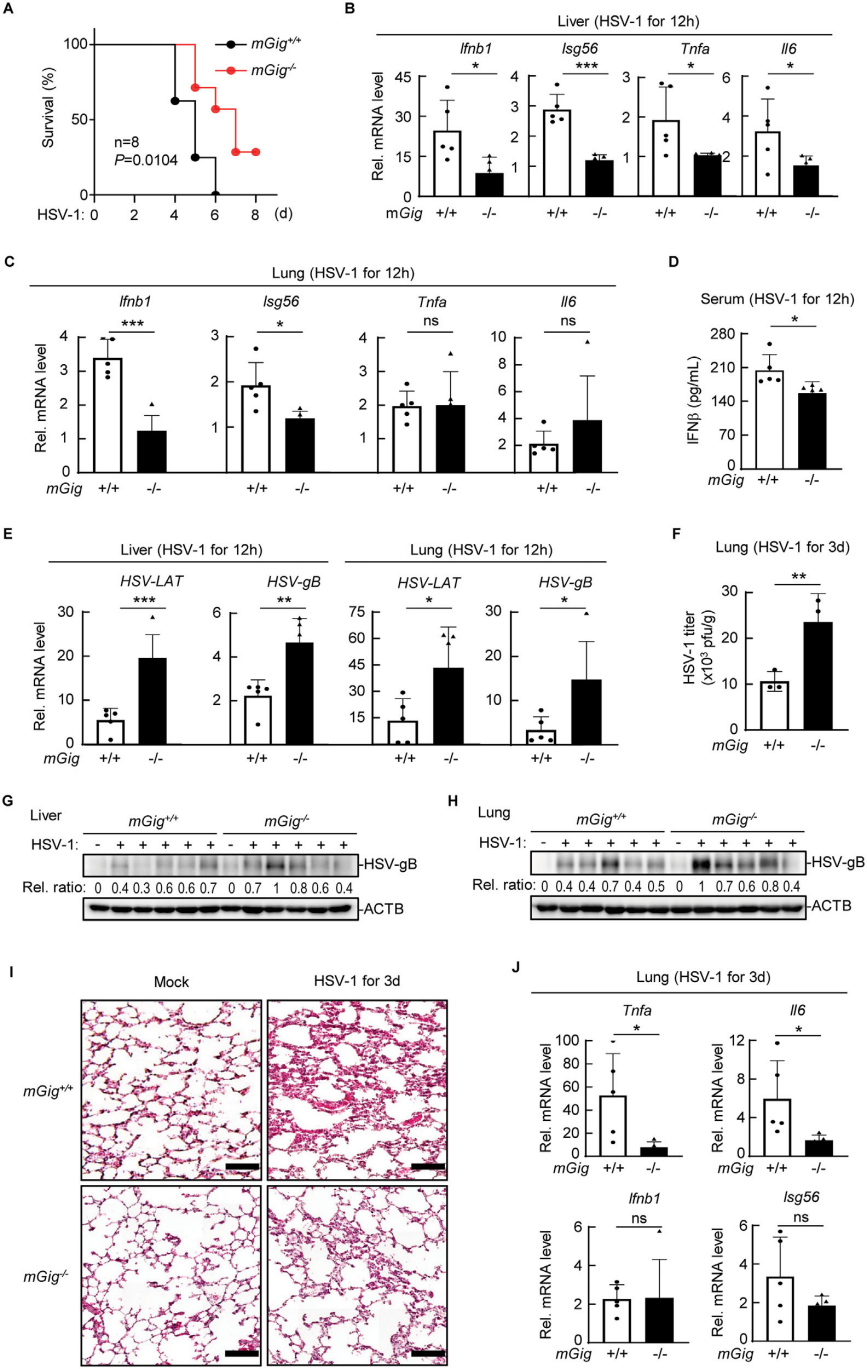
Gigaxonin is required for host defense against DNA viruses in mice. A) *mGig^+/+^* and *mGig^−/−^* mice (n  =  8 for each group) were intravenously injected with HSV‐1 (5×10^6^ PFU per mouse). Mouse survival was monitored, and the survival rates were calculated, presented as Kaplan–Meier curves. B–E) *mGig^+/+^* and *mGig^−/−^* mice (n  =  5 for each group) were injected with HSV‐1 for 12 h. The transcription of antiviral immune products in the livers (B) or lungs (C) of the mice was analyzed via qPCR as indicated. The sera of the mice were analyzed via ELISA (D). The transcription of viral genes in the livers or lungs of the mice was analyzed by qPCR as indicated (E). F) *mGig^+/+^* and *mGig^−/−^* mice (n  =  3 for each group) were injected with HSV‐1 for 3 days before lung tissue plaque assays were performed. G, H) *mGig^+/+^* and *mGig^−/−^* mice (n = 5 for each group) were injected with HSV‐1 for 12 h. The expression of the viral protein gB in the liver (G) and lungs (H) was analyzed by immunoblotting. The gB bands were quantified and normalized to the levels of the control ACTB. I,J) *mGig^+/+^* and *mGig^−/−^* mice (n  =  5 for each group) were injected with HSV‐1 for 3 days. Lung sections were stained with hematoxylin and eosin (I). Scale bars, 100 µm. The transcription of the indicated genes in mouse lungs was determined by qPCR (J). All bar graphs show the means ± SDs.^∗^ *p* < 0.05; ^∗∗^ *p* < 0.01; ^∗∗∗^ *p* < 0.001; ns, not significant.

To investigate the physiological role of gigaxonin in viral infection, we monitored the survival rates of *mGig* ^−/−^ and *mGig^+/+^* mice after intravenous injection of a lethal dose of HSV‐1 (5 × 10^6^ PFU per mouse). Compared with their wild‐type littermates, *mGig* ^−/−^ mice died later (5 days versus 4 days after infection) and had a greater survival rate (0/8 versus 3/8, *P* = 0.0104) (**Figure** **6**A). The transcription of *Ifnb1*, *Isg56*, *Tnfa*, and *Il6* in *mGig^−/−^* livers at 12 h after HSV‐1 injection was significantly lower than that in *mGig^+/+^* livers (Figure 6B). The transcription of *Ifnb1* and *Isg56* in *mGig^−/−^* lungs was similarly lower than that in *mGig^+/+^* lungs, but the transcription of *Tnfa* and *Il6* in the lungs was not significantly different between *mGig^−/−^ and mGig^+/+^* mice (Figure 6C). Consistent with the lower concentration of serum IFN‐β in *mGig* ^−/−^mice (Figure 6D), the transcription of viral *LAT* and *gB* in both *mGig* ^−/−^ liver and lung tissues was significantly higher than that in *mGig^+/+^* tissues (Figure 6E). The HSV‐1 titer in the *mGig* ^−/−^ mouse serum was also greater than that in the wild‐type control (Figure 6F). The immunoblotting assays also demonstrated that the expression of the viral protein gB in both the livers and lungs of *mGig* ^−/−^ mice was significantly greater than that in the corresponding tissues of *mGig^+/+^* mice (Figure 6G,H). Histopathological analysis 3 days after HSV‐1 injection revealed that HSV‐1 induced obvious inflammatory responses in the lung, characterized by thickened alveolar septa, infiltration of inflammatory cells, and dilation of capillaries (Figure 6I). Compared to those of the control lungs, the lungs of the *mGig* ^−/−^ mice exhibited significantly less immune cell infiltration and weaker inflammatory responses. Additionally, the transcription of *Tnfa* and *Il6*, but not *Ifnb1* and *Isg56*, in the lungs of *mGig* ^−/−^ mice was lower than that in the lungs of wild‐type mice (Figure 6J). These results suggest that gigaxonin depletion attenuates mouse antiviral immune responses and protects mice from virus‐induced inflammatory cytokine storms.

## 3 Discussion

In this study, we identified gigaxonin as a novel regulator that potentiates antiviral innate immune responses by targeting both TREX1 and cGAS. Biallelic variants in the gene encoding gigaxonin cause the hereditary disease giant axonal neuropathy because gigaxonin mediates the homeostasis of multiple neurofilaments via ubiquitination and proteasomal degradation.^[^ ^27^, ^29^, ^31^ ^]^ Gigaxonin also controls autophagosome formation in neurons by driving the ubiquitination and degradation of ATG16L1 through both the proteasomal and autophagy pathways.^[^ ^37^, ^38^ ^]^ Gigaxonin functions as a CRL3 substrate recognition module.^[^ ^27^, ^39^ ^]^ Previous studies have suggested that gigaxonin deficiency is not directly linked to inflammation or autoimmune diseases;^[^ ^40^, ^41^, ^42^ ^]^ however, our findings revealed that *gigaxonin* potentiates cGAS‐mediated antiviral signaling. *Gigaxonin* deficiency attenuates mouse antiviral immune responses and protects mice from death caused by HSV‐1 infection. Mechanistically, gigaxonin mediates the ubiquitination of TREX1 and cGAS through the CRL3 complex, contributing to the degradation of TREX1 and the activation of cGAS signaling.

TREX1 degrades excess self‐DNA and foreign DNA in the cytosol, thereby limiting aberrant activation of the cGAS pathway and downstream type I interferon responses.^[^ ^16^, ^22^, ^43^ ^]^ To date, only two distinct ubiquitination mechanisms have been reported to regulate the expression of TREX1. Wild‐type p53 transcriptionally upregulates the E3 ubiquitin ligase TRIM24, which triggers TREX1 ubiquitination and subsequent proteasomal degradation.^[^ ^44^ ^]^ An undefined E3 ligase catalyzes monoubiquitination of TREX1 at the K271 and K277 residues in its proximal C‐terminal region, leading to TREX1 relocating from the ER to autophagic vesicles or lysosomes.^[^ ^45^ ^]^ Our study revealed that the gigaxonin‐CRL3 complex is responsible for the ubiquitination and proteasomal degradation of TREX1 in the steady state. K242, K271, and K277 of TREX1 are responsible for gigaxonin‐mediated ubiquitination and degradation.

Due to the role of gigaxonin in TREX1 turnover, gigaxonin knockdown results in increased TREX1 expression, reduced cytosolic dsDNA levels, and subsequently impaired cGAS signaling, as we demonstrated in this study. However, when we performed dual‐luciferase reporter assays in HEK293T cells, in which TREX1 was not expressed,^[^ ^46^ ^]^ we found that gigaxonin potentiated cGAS‐MITA‐mediated activation of ISRE in a dose‐dependent manner, suggesting that gigaxonin can promote cGAS activation independently of TREX1. Our further experiments demonstrated that gigaxonin mediates the ubiquitination of cGAS via the CRL3 complex, thereby increasing the enzymatic activity of cGAS without affecting its expression.

The enzymatic activity of cGAS is regulated by DNA‐binding‐driven liquid‒liquid phase separation,^[^ ^36^, ^47^, ^48^ ^]^ posttranslational modifications,^[^ ^49^, ^50^, ^51^ ^]^ cGAS‐binding proteins, and ions.^[^ ^52^, ^53^ ^]^ Both cGAS and TREX1 can form phase‐separated liquid droplets in the presence of dsDNA in vitro.^[^ ^54^ ^]^ However, cGAS‒DNA liquid droplets restrict the entry and diffusion of TREX1 into the droplet center, which directly suppresses TREX1 exonuclease activity.^[^ ^54^ ^]^ Here, we reveal a novel mechanism by which both cGAS and TREX1 are regulated by gigaxonin. We demonstrated that gigaxonin interacts with both TREX1 and cGAS through its kelch repeats, and that cGAS attenuates the interaction between gigaxonin and TREX1 in a dose‐dependent manner. To support this conclusion from a structural perspective, we generated a structural interaction pattern among gigaxonin, TREX1 and cGAS via AlphaFold3 (GAN: NP_071324.1, TREX1: NP_338599.1 and cGAS: NP_612450.2). The structure prediction shows that the kelch repeats of gigaxonin form two 4‐stranded parallel β‐sheets. Between these two β‐sheets, a groove is formed into which the alpha‐helix of the C‐terminus of TREX1 inserts (Figure S4A,B; Supporting Information). However, gigaxonin interacts with cGAS through the exterior side of its kelch repeats (Figure S4C,D; Supporting Information). The N‐terminal intrinsically disordered region of cGAS covers the groove formed by two β‐sheets of gigaxonin, thereby spatially hindering the TREX1‐gigaxonin interaction. This structural analysis further revealed that endogenous gigaxonin interacts with TREX1 or cGAS in different states.

Because the substrate recognition module of CRL complexes is reported to be released after the ubiquitination of substrates,^[^ ^39^, ^55^ ^]^ we can deduce the dynamic roles of gigaxonin in regulating both TREX1 and cGAS: 1) In the steady state, gigaxonin regularly recognizes TREX1, mediating its proteasomal degradation by ubiquitination to maintain cellular homeostasis. 2) DNA virus infection or abnormal cytosolic dsDNA enhances gigaxonin dimerization, resulting in accelerated TREX1 turnover and the release of kelch repeats of gigaxonin. 3) Gigaxonin interacts with cGAS through its kelch repeats in an infection‐dependent manner, and subsequently enhances cGAS ubiquitination and enzymatic activity. The binding of cGAS with gigaxonin blocks gigaxonin‐TREX1 interaction.

In conclusion, our study identified gigaxonin as a dual regulator of antiviral innate immune responses and elucidated a dynamic modulatory mechanism mediated by gigaxonin‐dependent ubiquitination of either TREX1 or cGAS. Given the critical roles of cGAS and TREX1 in multiple diseases, especially type I interferonopathies, our findings suggest that targeting gigaxonin may provide a novel therapeutic strategy for combating diseases related to the TREX1‐cGAS pathway.

## 4 Experimental Section

### Animals and Ethics Statement

*Ethics Statement*: All the mice were housed in a specific pathogen‐free animal facility at Wuhan University. All authentic viral studies were approved by the Animal Care and Use Committee of Wuhan University (protocol number WP20210527) and were performed in the animal biosafety level 2 laboratory at Wuhan University (SKLV‐AE2023 012).

C57BL/6 *gigaxonin* ^−/−^ mice (strain NO. T030141) were purchased from GemPharmatech Co., Ltd. (Nanjing, China). Male *gigaxonin* ^+/+^ and *gigaxonin* ^−/−^ mice (8–10 weeks old) were intravenously injected with HSV‐1 (ATCC VR‐1789, 5 × 10^6^ PFU per mouse), and the survival of the mice was monitored every day. Mouse serum, lung, and liver tissues were collected for ELISA, qPCR, or histopathological analysis at 12 h or 3 days after infection, respectively.

### Plasmid Constructs

Expression plasmids for gigaxonin, cGAS, TREX1 and their mutants were constructed via standard molecular biology techniques. The shRNAs and sgRNAs were synthesized and inserted into the pLKO.1 (Addgene, 10878) and LentiCRISPR v2 (Addgene, 52961) vectors. The sequences of the shRNA and sgRNA were as follows: shCon, 5′‐CCGCAGGTATGCACGCGT‐3′; shGig#1, 5′‐GCAAGACATAACTTCGGAATT‐3′; shGig#2, 5′‐CCGTGACTTTGCACTACATTA‐3′; sgCon, 5′‐GACCAGGATGGGCACCACCC‐3′; sgGig#1, 5′‐GCAGAAGAACATCCTGGCGG ‐3′; sgGig#2, 5′‐AGCGCTCAGCTCTTTCCGCG‐3′.

### Stable Cell Line Generation

The packaging plasmids psPAX2 (Addgene, 12260) and pMD2. G (Addgene, 12259) were cotransfected with pLKO‐shRNA or lentiCRISPR‐sgRNA plasmids into HEK293T cells. Two days later, culture medium containing lentiviruses was collected and applied to THP‐1 or HeLa cells in the presence of 8 µg mL^−1^ polybrene (Millipore, TR‐1003). The infected cells were selected with 1 µg mL^−1^ puromycin (Med ChemExpress) for 7 days before additional experiments.

### Virus and Plaque Assays

HSV‐1 (ATCC VR‐1789), VACV (Tian‐Tan strain) and MCMV (ATCC CRL‐1404) strains were obtained from the China Center for Type Culture Collection. For plaque assays, the cells were infected with HSV‐1 for 16 h. The supernatants of cultured cells were serially diluted and added to Vero cells (ATCC CCL‐81) for 2 h, followed by a 2% methylcellulose overlay. After 2 days, Vero cells were fixed with 4% paraformaldehyde (Biosharp, BL539A) for 30 min and stained with 1% crystal violet (HUSHI, 71012314) before plaque counting.

### Co‐Immunoprecipitation and Immunoblotting Analysis

The cells were lysed in Nonidet P‐40 lysis buffer (1 mm EDTA, 150 mm NaCl, 1% Nonidet P‐40, 20 mm Tris‐HCl, pH 7.4) supplemented with a protease inhibitor cocktail (Selleckchem, B14001). After centrifugation at 12000 × g for 10 min at 4 °C, the supernatant was incubated with the indicated antibody or control IgG and Protein G Sepharose for 2 h. Sepharose beads were washed three times with 1 mL of lysis buffer containing 0.5 m NaCl. The precipitates were analyzed via standard immunoblotting procedures. The information for the antibodies and small molecules used in the study can be found in the supplementary Tables.

### RNA Isolation and Quantitative Real‐time PCR (qPCR)

The MIQE 2.0 guidelines was followed.^[^ ^56^ ^]^ Total RNA was extracted from cells or tissues using RNAiso Plus (TaKaRa, 9109) and then reverse‐transcribed into cDNA with ToloScript RT EasyMix (TOLOBIO, 22107). The cDNA was subjected to real‐time PCR analysis to measure the expression of mRNA. The gene‐specific sequences of primers used were as described previously ^[^ ^36^, ^57^ ^]^ or as follows: human *gigaxonin* (forward: 5′‐TCTGAGTGCATCGTGACTGTT‐3′; reverse: 5′‐CCCTCTCTATGACCCTAACAGG‐3′); and human *TREX1* (forward: 5′‐GCATCTGTCAGTGGAGACCA‐3′; reverse: 5′‐AGATCCTTGGTACCCCTGCT‐3′).

### Dual‐Luciferase Reporter Assay

The cells were seeded and transfected the following day via the standard calcium phosphate precipitation method. Empty control plasmids were added to ensure that each transfection received the same total amount of DNA. To normalize for transfection efficiency, pRL‐TK *Renilla* luciferase reporter plasmids were added to each transfection. The ISRE‐luciferase reporter plasmid was purchased from Stratagene (La Jolla, CA). Luciferase assays were performed via a dual‐specific luciferase assay kit (Promega, E1910). Firefly luciferase activities were normalized on the basis of *Renilla* luciferase activities.

### Purification of the Recombinant Proteins

The pET28a‐6×His‐SUMO‐cGAS and pET28a‐6×His‐SUMO‐gigaxonin‐ΔLink plasmids were expressed in *E. coli* BL21 cells. After IPTG (Solarbio, I8070) treatment for 16 h, the bacterial cells were collected and lysed with lysis buffer (10 mm imidazole, 400 mM NaCl, and 10% glycerol in 20 mM Tris‐HCl, pH 7.9) by high‐pressure crushing. After centrifugation at 12 000 × g for 10 min at 4 °C, the His‐SUMO‐tagged cGAS or gigaxonin‐ΔLink proteins in the supernatant were purified with *Ni‐NTA*‐Sepharose beads (Smart‐Life Sciences, SA036025) and eluted with imidazole. The untagged proteins were obtained by incubating 6×His‐SUMO‐cGAS with (ULP, followed by Ni^2+^ affinity chromatography.

### Measurement of cGAS Activity In Vitro

The measurement of cGAS‐synthesized pyrophosphate in vitro was described previously.^[^ ^36^ ^]^ Briefly, recombinant cGAS (2.5 µm) and/or gigaxonin‐ΔLink (0.5, 1, and 2.5 µm) were mixed with buffer (5 mm MgCl2, 2 mm ATP, 2 mm GTP, 0.2 mg mL^−1^ BSA and 0.25 U mL^−1^ pyrophosphatase (Beyotime, R7025S) in 20 mM HEPES, pH 7.5). The mixture was incubated with or without HT‐DNA (100 ng mL^−1^) at 37 °C for 1 h, after which malachite green (Beyotime, S0196S) was added. The intensity of the end‐product molybdophosphoric acid complex, which reflects the cGAS enzymatic reaction, was measured at 620 nm.

### Statistics and Reproducibility

The experimental data from the cells are representative of two or more independent experiments with similar results. The bar graphs show the means ± standard deviations (SDs) derived from three replicates of a representative experiment unless specified otherwise in the figure legends, and *P* values were calculated via two‐tailed unpaired Student's t tests via GraphPad Prism 8. The number of experimental animals (n) is indicated in the figure legends.

### Statements

All the authors agree with the submission.

The work has not been published elsewhere, either completely or in part or any other form or language.

If material has been reproduced from another source, the authors have authorization from the copyright holder (usually the Publisher) to use it and have included this authorization with their submission.

The authors declare that they have no competing financial interests.

Animal experiments were approved by national or local authorities.

Experiments do not involve patients or clinical trial issues.

## Conflict of Interest

The authors declare no conflict of interest.

## Author Contributions

Y.L. and B.Z.J. designed the research; B.Z.J., J.W., Y.T., S.Z., Y.S.W., Q.Q.Z., and H.M.H. performed the experiments; B.Z.J., Y.S.W., Y.H., and B.Z. critically discussed the results. B.Z.J. and Y.T. wrote the manuscript; Y.L. supervised the project and edited the manuscript. Y.L. acquired funding and managed the project.

## Supporting information



Supporting Information

## Data Availability

The data that support the findings of this study are available from the corresponding author upon reasonable request.
