# Supplementary material for: Gigaxonin Potentiates Antiviral Innate Immune Responses by Targeting cGAS and TREX1
Source: Adv Sci (Weinh). 2025 Sep 11;12(44):e07146. doi: 10.1002/advs.202507146 (PMC12667517; doi:10.1002/advs.202507146)
Supplement: Supplementary file 1 — Supporting Information [file ADVS-12-e07146-s001.pdf]

Figure S1

A

GENE_NAME	Protein IDs	Peptides	Razor + unique peptides	Unique peptides	Sequence coverage [%]	Unique + razor sequence coverage [%]	Unique sequence coverage [%]	Mol. weight [kDa]
cGAS	Q8C6L5	20	20	20	40.2	40.2	40.2	58.193
Gigaxonin	Q8CA72	13	13	13	26.8	26.8	26.8	67.67

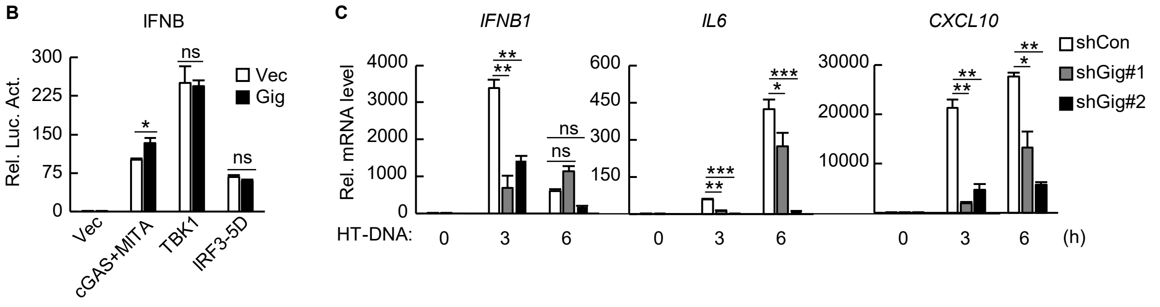

A) Original information on the identification of mouse Gig as a mouse cGAS-associated protein. **B)** HEK293T cells were transfected with the IFNB luciferase reporter (0.1 μg/mL), TK-Renilla luciferase reporter (0.01 μg/mL), and the indicated plasmids (0.1 μg/mL each). Luciferase assays were performed 20 hours after transfection. **C)** Stable Gig knockdown and control THP-1 cells were transfected with HT-DNA (1 mg/mL) for the indicated times before qPCR analysis. The bar graphs show the mean ± S.D. (n=3) of a representative experiment performed in triplicate. *P < 0.05, **P < 0.01, ***P < 0.001. ns, not significant.

Figure S2

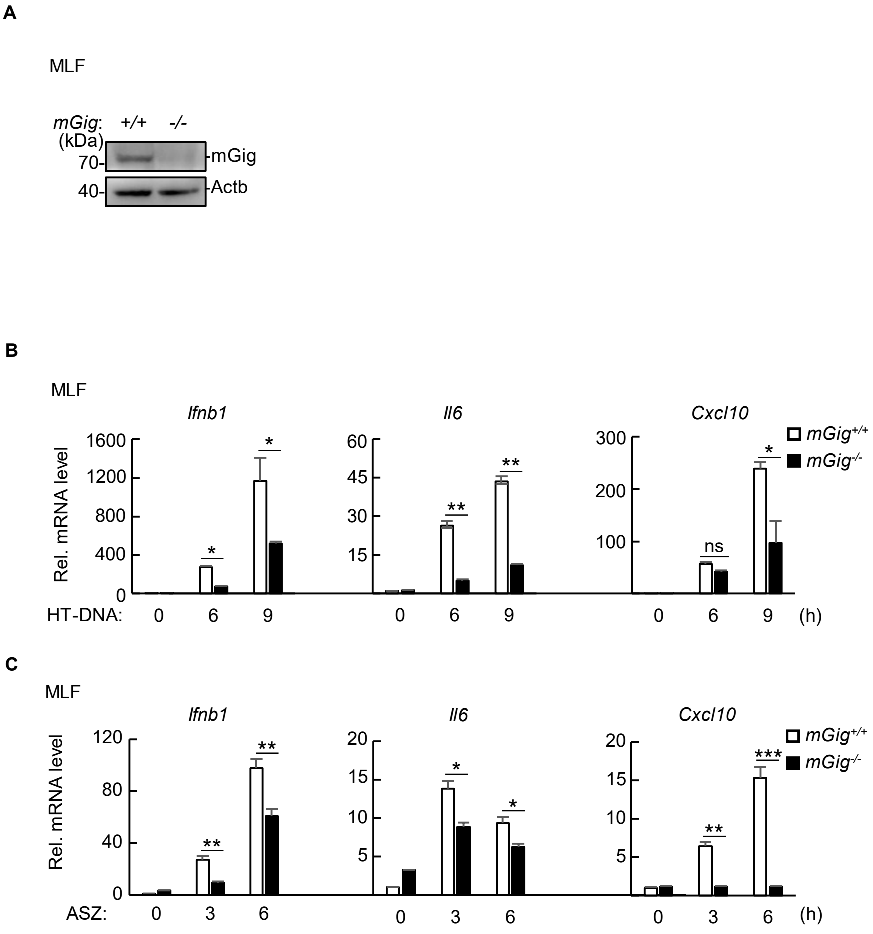

A) The knockout efficiency of mouse Gig in primary MLFs was analyzed by immunoblotting. **B, C)** $Gig^{+/+}$ and $Gig^{-/-}$ MLFs were transfected with HT-DNA (1 mg/mL) (**B**) or treated with ABT737 (5 μM),S63845 (5 μM), and Z-VAD (5 μM) (**C**) for the indicated times before qPCR analysis. The bar graphs show the mean ± S.D. (n=3) of a representative experiment performed in triplicate. *P < 0.05, **P < 0.01, ***P < 0.001. ns, not significant.

Figure S3

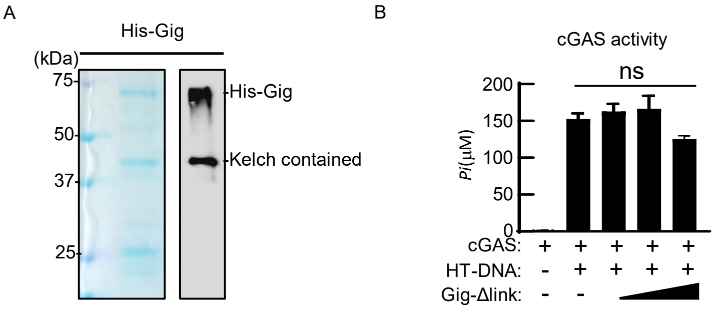

A) Recombinant His-tagged Gig-protein was analyzed by coomassie blue staining (left) or immunoblotting (right). **B)** Recombinant cGAS and dose-dependent Gig-Δlink protein were incubated with HT-DNA (100 ng/mL), ATP, and GTP. Synthesized inorganic pyrophosphates (PPi) were measured with the indicated kit. The bar graphs show the mean ± S.D. (n=3) of a representative experiment performed in triplicate. ns, not significant.

Figure S4

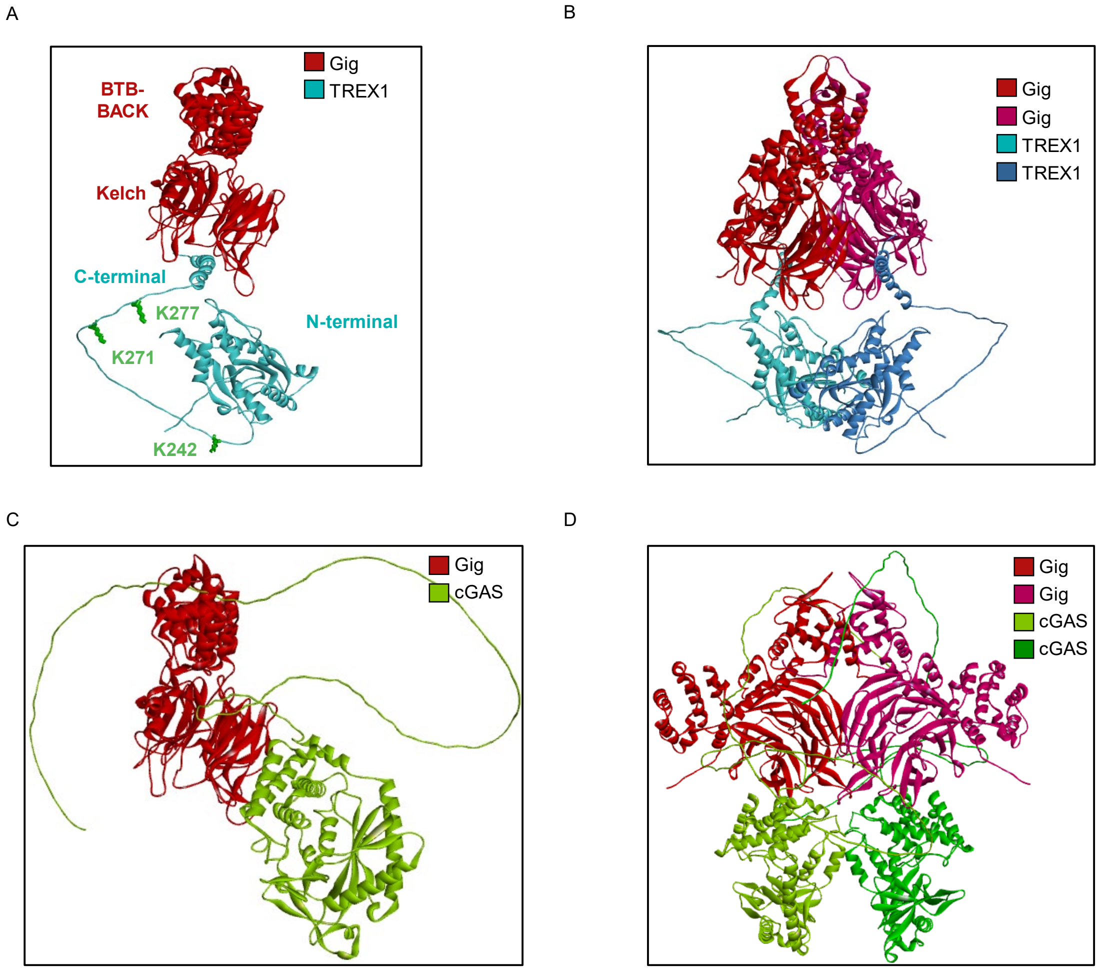

A,B,C,D) The interaction structural model of Gig-TREX1 (**A**), Gig/TREX1 dimer (**B**), Gig-cGAS (**C**) and Gig/cGAS dimer (**D**) was predicted by AlphaFold3 and visualized using the Discovery Studio Visualizer.

Table S1 Small molecules used in this study

Drug/inhibitor	Company	Catalogue number
ATP	Sigma–Aldrich	A1852
GTP	Sigma–Aldrich	G8877
Digitonin	Sigma–Aldrich	D141
ABT-737	MedChemExpress	HY-50907
S63845	MedChemExpress	HY-100741
Z-VAD-FMK	MedChemExpress	HY-16658B
MG132	Selleck	S2619

Table S2 Antibody used in this study

Antibody	Supplier	Catalog number
Mouse anti-HA monoclonal antibody	ORIGENE	TA180128
Mouse anti-Flag monoclonal antibody	ORIGENE	TA50011-100
Rabbit anti-Myc monoclonal antibody	Abclonal	AE070
Rabbit anti-ACTB monoclonal antibody	Abclonal	AC026
Rabbit anti-IRF3 monoclonal antibody	Abclonal	A19717
Rabbit anti-ubiquitin monoclonal antibody	Abclonal	A19686
Rabbit anti-phospho-IRF3 (Ser386) monoclonal antibody	HUABIO	ET1608-22
Rabbit anti-NAK/TBK1 monoclonal antibody clone EPR2867(2)	Abcam	ab109735
Rabbit anti-phospho-NAK/TBK1 (Ser172) monoclonal antibody clone EPR2867(2)	Abcam	ab109272
Mouse anti-pIkba monoclonal antibody	CST	#9246
Rabbit anti- phospho-IRF3(Ser396) monoclonal antibody	CST	#4947
Rabbit anti-cGAS monoclonal antibody	CST	#83623
Rabbit anti-Ikba Polyclonal antibody	Proteintech	10268-1-AP
Rabbit anti-TREX1 monoclonal antibody	Proteintech	24876-1-AP
Mouse anti-Gigaxonin monoclonal antibody	Santa Cruz Biotechnology	sc-376173
